# Prognosis of IGLV3-21^R110^ chronic lymphocytic leukemia after chemotherapy-based treatment in a real-world analysis

**DOI:** 10.1038/s41375-023-01975-0

**Published:** 2023-07-21

**Authors:** Paul J. Hengeveld, Hendrik Veelken, Cornelis A. M. van Bergen, Edwin Quinten, Mischa Y. L. Vervoordeldonk, Wahija Ismailzada, Rob S. Barendse, Julie M. N. Dubois, Marinus H. J. van Oers, Christian H. Geisler, Arnon P. Kater, Peter E. Westerweel, Anton W. Langerak, Mark-David Levin

**Affiliations:** 1 Department of Immunology, Erasmus MC, University Medical Centre Rotterdam, Rotterdam, the Netherlands; 2 Department of Internal Medicine, Albert Schweitzer Hospital, Dordrecht, the Netherlands; 3 Department of Hematology, Leiden University Medical Center, Leiden, the Netherlands; 4 Department of Hematology, Cancer Care Center Amsterdam, Amsterdam University Medical Centers, Amsterdam, the Netherlands; 5 Department of Hematology, Rigshospitalet, Copenhagen, Denmark

**Keywords:** Chronic lymphocytic leukaemia, Chronic lymphocytic leukaemia

## To the Editor:

Distinctive molecular patterns in the clonotypic B-cell receptor (BCR) classify patients with chronic lymphocytic leukemia (CLL) into immunogenetic subsets with consistent clinicobiological profiles [1]. Around 15–25% of all CLL patients are characterized by a clonotypic immunoglobulin (IG) light chain rearrangement using IGLV3-21*01/*04, featuring a stereotyped somatic hypermutation (G110R) at the IGLJ-IGLC border site (IGLV3-21^R110^) [2–4]. Patients with IGLV3-21^R110^ CLL present more frequently with advanced disease and have short time to first treatment (TTFT) and overall survival (OS) [2]. However, the predictive impact of the IGLV3-21^R110^ genotype is less clearly understood. In a recent analysis of two randomized trials that evaluated the efficacy of targeted agents, we did not observe differences in the response, minimal residual disease depth or progression-free survival (PFS) of IGLV3-21^R110^ patients, compared to all other patients, suggesting that targeted agents mitigate the adverse risk associated with IGLV3-21^R110^ CLL [4]. In addition, a pooled analysis of trials demonstrated that, irrespective of their IGHV mutational status, patients with subset #2 CLL, which invariably carry an IGLV3-21^R110^ IG light chain, have shorter time to next treatment (TTNT) after chemoimmunotherapy-based treatment [5]. However, to determine whether all IGLV3-21^R110^ CLL patients should, irrespective of IG heavy chain stereotypy, preferentially receive targeted agents, characterization of the predictive impact of the IGLV3-21^R110^ genotype in the context of chemotherapy-based treatment is pivotal.

Here, we assess the real-world prognosis of patients with IGLV3-21^R110^ CLL after first-line chemotherapy-based treatment. Patient samples were obtained from the biobank of the Leiden University Medical Center (*n* = 140), the Academic Medical Center Amsterdam Biobank for B cell malignancies (*n* = 25) and the HOVON-68 databank (*n* = 55), all approved by the respective institutional ethical review boards [6]. All patients provided informed consent. The clonotypic IG light chain sequence was determined previously [2, 4]. All patients provided informed consent and all TTNT was defined as the interval between the first day of index therapy and the first day of a subsequent line of anti-leukemic therapy or Richter’s transformation, while OS was defined as the interval between the first day of index therapy and death. Survival analysis was performed using Kaplan–Meier estimation in *R*. Statistical significance was evaluated using omnibus and pairwise log-rank testing.

IG light chain sequencing and follow-up up data were available for 219/220 CLL patients. One patient, having received ibrutinib as first-line treatment, was excluded from the analysis. Patients were diagnosed between 1983–2019 and were treated between 1986–2022. The median follow-up time was 122 months. Baseline characteristics of the 218 patients are listed in Table 1. The IGLV3-21^R110^ genotype was present in 35/218 (16%) patients. Baseline demographics and the IGHV mutational status were well-balanced between the groups. In agreement with previous publications, trisomy 12 and del(17p) were exclusively present in patients without IGLV3-21^R1102–4^.

**Table 1:** Baseline characteristics of the cohort, stratified by light chain genotype.

	IGLV3-21^R110^ (*n* = 35)	Other light chain (*n* = 183)
Sex (*n*, %)		
Male	26 (74%)	125 (68%)
Female	9 (26%)	58 (32%)
Age, years (median, IQR)	60 (52–66)	59 (51–67)
RAI stage at diagnosis (*n*, %)		
0	7 (20%)	49 (27%)
I	9 (26%)	26 (14%)
II	5 (14%)	11 (6%)
III	0 (0%)	3 (2%)
IV	2 (6%)	7 (4%)
Missing	12 (34%)	87 (47%)
IGHV mutational status (*n*,%)		
Unmutated	18 (51%)	107 (58%)
Mutated	16 (46%)	69 (38%)
Missing	1 (3%)	7 (4%)
Cytogenetic abs. (*n*,%)		
Del13q14	21 (60%)	55 (30%)
Del11q22	2 (6%)	18 (10%)
Trisomy 12	0 (0%)	26 (14%)
Del17p	0 (0%)	15 (8%)
Missing	2 (6%)	49 (27%)
First-line treatment (*n*, %)		
None	2 (6%)	32 (17%)
Chemo(immuno)-based		
Chlorambucil only	10 (31%)	51 (28%)
FCR	5 (14%)	24 (13%)
FC	4 (11%)	26 (14%)
BR	2 (6%)	2 (1%)
R-CVP	1 (3%)	2 (1%)
CVP	1 (3%)	2 (1%)
R-Chlorambucil	3 (9%)	8 (4%)
O-Chlorambucil	1	2 (1%)
R-Chorambucil-Len	1 (3%)	2 (1%)
Fludarabine only	0 (0%)	2 (1%)
R-CHOP	0 (0%)	1 (1%)
* Other*		
Prednisone	0 (0%)	1 (1%)
Radiotherapy	0 (0%)	1 (1%)
FCA	5 (14%)	26 (14%)
VMP	0 (0%)	1 (1%)
Alive at last observation (*n*, %)		
Yes	16 (46%)	98 (54%)
No	19 (54%)	85 (46%)

*abs* aberrations, *BR* bendamustine and rituximab, *CVP* cyclophosphamide, vincristine and prednisone, *FC* fludarabine and cyclophosphamide, *FCA* fludarabine, cyclophosphamide and alemtuzumab, *FCR* fludarabine, cyclophosphamide and rituximab, *IQR* interquartile range, *O-Chlorambucil* obinutuzumab and chlorambucil, *R-Chlorambucil* rituximab and chlorambucil, *R-Chlorambucil-Len* rituximab, chlorambucil and lenalidomide, *R-CHOP* rituximab, cyclophosphamide, hydroxydaunomycine, vincristine and prednisone, *R-CVP* rituximab, cyclophosphamide, vincristine and prednisone, *VMP* bortezomib, melphalan and prednisone.

At time of data review, 33/35 (94%) of IGLV3-21^R110^ patients and 151/183 (83%) patients with any other light chain had progressed to therapy. As previously reported [2], TTFT was markedly shorter in patients with IGLV3-21^R110^ CLL, compared to CLL with mutated IGHV (M-CLL) (median TTFT 31.9 months [95% CI: 21.0–48.0] versus 183.0 months [95% CI: 121.1-NR], *P* < 0.0001). Indeed, median TTFT of IGLV3-21^R110^ patients was more similar to patients with U-CLL (U-CLL: 14.3 months, 95% CI: 9.7–28.5, *P* = 0.06). First-line treatment regimens were comparable in both groups, with most patients having received chlorambucil monotherapy (Table 1).

**Fig. 1:**
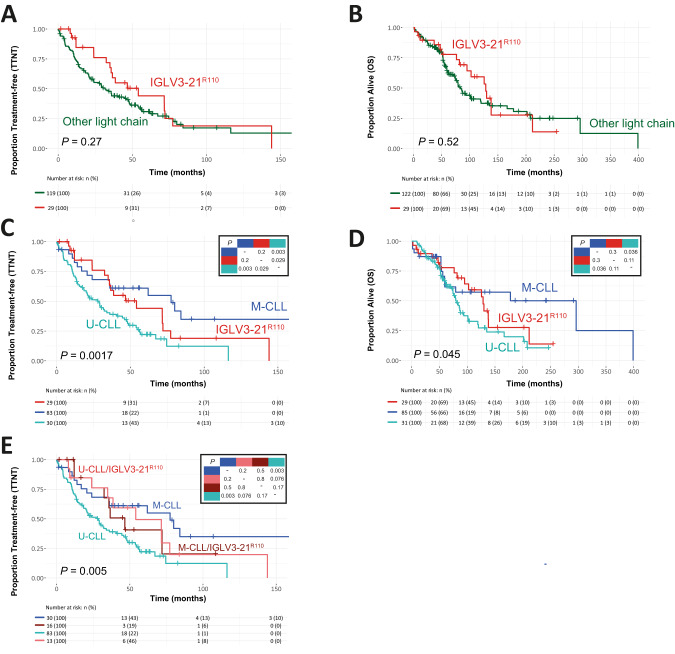
Efficacy of chemotherapy-based treatment for IGLV3-21^R110^ CLL in a real-world setting. Kaplan–Meier survival curves and risk tables, indicating time to next treatment (**A**, **C**, **E**) or overall survival (**B**, **D**), stratified per IG light chain genotype and/or IGHV mutational status. An asterisk indicates a censoring event. *P*-values (lower left) were calculated using an omnibus log-rank test. The top right panel indicates head-to-head *P*-values, calculated using a log-rank test. All figures indicate survival following treatment with any chemo(immuno)therapy-based regimen. Abbreviations: M-CLL CLL with mutated IGHV; M-CLL/IGLV3-21^R110^, CLL with mutated IGHV and IGLV3-21^R110^; OS overall survival; TTNT time to next treatment; U-CLL, CLL with unmutated IGHV, U-CLL/IGLV3-21^R110^; CLL with unmutated IGHV and IGLV3-21^R110^.

Notably, when including only patients that received chemotherapy-based treatment, stratification by IGLV3-21^R110^ genotype did not reveal significant differences in TTNT and OS (Fig. 1A, B) (IGLV3-21^R110^ vs any other light chain, median TTNT: 54.1 months [95% CI: 35.7-NR] vs 31.6 months [95% CI: 22.0–47.8], *P* = 0.3, median OS: 128.4 months [95% CI: 95.1-NR] vs 83.6 months [74.2–120.0], *P* = 0.5). When stratifying patients without IGLV3-21^R110^ by IGHV mutational status, IGLV3-21^R110^ CLL patients had significantly longer TTNT, but not OS, compared to patients with unmutated IGHV (U-CLL) (median TTNT 54.1 months [95%CI 35.7 -NR] versus 27.8 [18.5–44.6] months, *P* = 0.028, median OS 128.4 months [95%CI 95.1-NR] versus 80.1 months [71.7–101.0], *P* = 0.11), but not compared to patients with M-CLL (median TTNT 54.1 months [95%CI 35.7 -NR] versus 77.5 months [95% CI: 35.7-NR], *P* = 0.2, median OS 128.4 months [95% CI: 95.1-NR] versus 296.2 months [95% CI: 59.3-NR], *P* = 0.3) (Fig. 1C, D). Within the IGLV3-21^R110^ patient group, there were no significant differences in TTNT between patients with unmutated (U-IGLV3-21^R110^, n = 13) and mutated IGHV (M-IGLV3-21^R110^, n = 16) (54.1 months [95%CI 34.9-NR] versus 46.6 months [95%CI 32.4-NR], *P* = 0.8) (Fig. 1E). Whereas there was no difference in median TTNT between M-IGLV3-21^R110^ and M-CLL with other light chains (46.6 months [95% CI: 32.4-NR] vs 77.5 [95% CI: 35.7-NR], *P* = 0.5), the difference between U-IGLV3-21^R110^ and U-CLL with any other light chain approached statistical significance (54.1 months [95% CI: 34.9-NR] versus 27.8 months [95% CI: 18.5–44.6], *P* = 0.076) (Fig. 1E). As there were very few events per arm, a similarly stratified approach for OS was not feasible. In a separate analysis, including only patients receiving chlorambucil monotherapy, similar patterns were apparent, with IGLV3-21^R110^ signifying a group of patients with intermediate TTNT and OS, compared to patients with U-CLL and M-CLL (Supplementary Fig. 1). However, none of these differences reached statistical significance.

Due to a constrained cohort size and treatment heterogeneity, statistical power was limited in this real-world analysis. Furthermore, the most frequently used treatment regimen in this group, chlorambucil monotherapy, is no longer the recommended standard-of-care for the treatment of CLL. In addition, clinical response and PFS could not be reliably estimated due to irregular reporting of international workshop on CLL (iwCLL) criteria. Finally, as this analysis was retrospective in nature, confounding by indication cannot be fully excluded. That said, the documented baseline characteristics indicated that the cohorts are comparable.

In summary, these data suggest that in a real-world setting, IGLV3-21^R110^ CLL patients may, irrespective of their IGHV mutational status, have longer TTNT after chemotherapy-based treatment, compared to patients with U-CLL. This implies that IGLV3-21^R110^ may represent a mainly prognostic, but not predictive marker, signifying CLL with a short indolent phase that nevertheless responds favorably to both novel agents and chemotherapy-based treatment. The importance of classical stratification by IGHV mutational status in patients with IGLV3-21^R110^ CLL seems limited. Based on these intriguing observations, the question whether chemo(immuno)therapeutic and targeted agents are equally efficacious in IGLV3-21^R110^ CLL warrants further exploration in a more controlled setting, preferably in a randomized trial.

## Supplementary information

Supplementary figure legend

Supplementary figure 1

## Data Availability

Data will be made available upon reasonable request to the corresponding author.
