# Supplementary material for: Prognosis of IGLV3-21R110 chronic lymphocytic leukemia after chemotherapy-based treatment in a real-world analysis
Source: Leukemia. 2023 Jul 21;37(9):1929–32. doi: 10.1038/s41375-023-01975-0 (PMC10457177; doi:10.1038/s41375-023-01975-0)
Supplement: Supplementary file 1 — Supplementary figure legend [file 41375_2023_1975_MOESM1_ESM.docx]

*Supplementary Figure 1. Efficacy of chlorambucil monotherapy for IGLV3-21^R110^ CLL in a real-world setting.*

Kaplan-Meier survival curves and risk table, indicating time to next treatment (1A) or overall survival (1B), stratified per IG light chain genotype and IGHV mutational status, following treatment with chlorambucil monotherapy. An asterisk indicates a censoring event. *P*-values (lower left) were calculated using an omnibus log-rank test. The top right panel indicates head-to-head *P*-values, calculated using a log-rank test

Abbreviations: M-CLL, CLL with mutated IGHV; TTNT, time to next treatment; U-CLL, CLL with unmutated IGHV.
